# Pharmacogenomic identification of small molecules for lineage specific manipulation of subventricular zone germinal activity

**DOI:** 10.1371/journal.pbio.2000698

**Published:** 2017-03-28

**Authors:** Kasum Azim, Diane Angonin, Guillaume Marcy, Francesca Pieropan, Andrea Rivera, Vanessa Donega, Claudio Cantù, Gareth Williams, Benedikt Berninger, Arthur M. Butt, Olivier Raineteau

**Affiliations:** 1 Brain Research Institute, University of Zürich/ETHZ, Zürich, Switzerland; 2 Adult Neurogenesis and Cellular Reprogramming, Institute of Physiological Chemistry, University Medical Centre of the Johannes Gutenberg University Mainz, Germany; 3 Focus Program Translational Neuroscience, Johannes Gutenberg University Mainz, Germany; 4 Univ Lyon, Université Claude Bernard Lyon 1, Inserm, Stem Cell and Brain Research Institute U1208, Bron, France; 5 School of Pharmacy and Biomedical Sciences, University of Portsmouth, Portsmouth, United Kingdom; 6 IMLS, University of Zurich, Zurich, Switzerland; 7 Wolfson Centre for Age-Related Diseases, King's College London, Guy's Campus, London, United Kingdom; NIMH/NIH, United States of America

## Abstract

Strategies for promoting neural regeneration are hindered by the difficulty of manipulating desired neural fates in the brain without complex genetic methods. The subventricular zone (SVZ) is the largest germinal zone of the forebrain and is responsible for the lifelong generation of interneuron subtypes and oligodendrocytes. Here, we have performed a bioinformatics analysis of the transcriptome of dorsal and lateral SVZ in early postnatal mice, including neural stem cells (NSCs) and their immediate progenies, which generate distinct neural lineages. We identified multiple signaling pathways that trigger distinct downstream transcriptional networks to regulate the diversity of neural cells originating from the SVZ. Next, we used a novel in silico genomic analysis, searchable platform-independent expression database/connectivity map (SPIED/CMAP), to generate a catalogue of small molecules that can be used to manipulate SVZ microdomain-specific lineages. Finally, we demonstrate that compounds identified in this analysis promote the generation of specific cell lineages from NSCs in vivo, during postnatal life and adulthood, as well as in regenerative contexts. This study unravels new strategies for using small bioactive molecules to direct germinal activity in the SVZ, which has therapeutic potential in neurodegenerative diseases.

## Author summary

The subventricular zone (SVZ) is the largest germinal zone of the postnatal and adult brain. It contains neural stem cells (NSCs) that give rise to neurons and oligodendrocytes (OLs) in a region-specific manner. Here, we use a bioinformatics approach to identify multiple signaling pathways that regulate the diversity of cell lineages that originate from different subregions of the SVZ. We further use a computational-based drug-discovery strategy to identify a catalogue of small molecules that can be used to manipulate the regionalization of the SVZ. We provide proof that, by administration of small molecules in vivo, it is possible to promote the specific generation of neurons and OLs from NSCs in both the postnatal and adult brain, as well as in regenerative contexts after lesion. This study unravels novel strategies for using small bioactive molecules to direct germinal activity in the SVZ, which has therapeutic potential in neurodegenerative diseases.

## Introduction

Controlling the fate of neural stem cells (NSCs) is a key therapeutic strategy in neuroregenerative medicine. The most promising and direct approach would be to use small molecules to promote the generation of a particular neural lineage, without the need to introduce complex genetic methods. A novel strategy consists of identifying drug-like compounds with the ability to induce transcriptional changes that are similar to those observed within neurogenic niches and are associated with acquisition of a specific cell fate [1,2]. Such a strategy is facilitated by the accumulation of publicly available datasets that allows the systematic comparison and identification of similarities between transcriptional signatures of biological and drug-induced samples [3], a principle that lies behind the connectivity map (CMAP) project [4]. In the adult and postnatal brain, neurogenesis is largely restricted to the subventricular zone (SVZ) of the lateral ventricle and the dentate gyrus of the hippocampal formation [5,6]. Within the SVZ, NSCs generate both neuronal precursors (NPs) and oligodendrocyte (OL) precursors (OPs) throughout life in a region-dependent manner [6,7]. Subsequently, NPs and OPs migrate to their final sites in the brain, where they differentiate, respectively, into neurons and OLs. Hence, directing fate of NSCs in the SVZ is a key therapeutic strategy for promoting repair following neurodegeneration or demyelination.

The NSCs of the postnatal SVZ are heterogeneous, both in terms of embryonic origins and of the distinct neural subtypes they generate depending on their spatial location [8,9]. This regional NSC heterogeneity is controlled by multiple extrinsic and intrinsic factors that could be exploited for therapeutic manipulation [6,7,10–12]. In the present study, we have determined links between the signaling pathways and transcriptional networks that define NSC lineages in the SVZ and we have identified small molecules that target them to regulate cell fate in vivo. Our findings facilitate the control of oligodendroglial and neuronal lineages in the postnatal and adult brain and offer new means to fully exploit the regenerative potential of the SVZ. 

## Results

### Identification of divergent signaling pathways in SVZ microdomains

**Fig 1:**
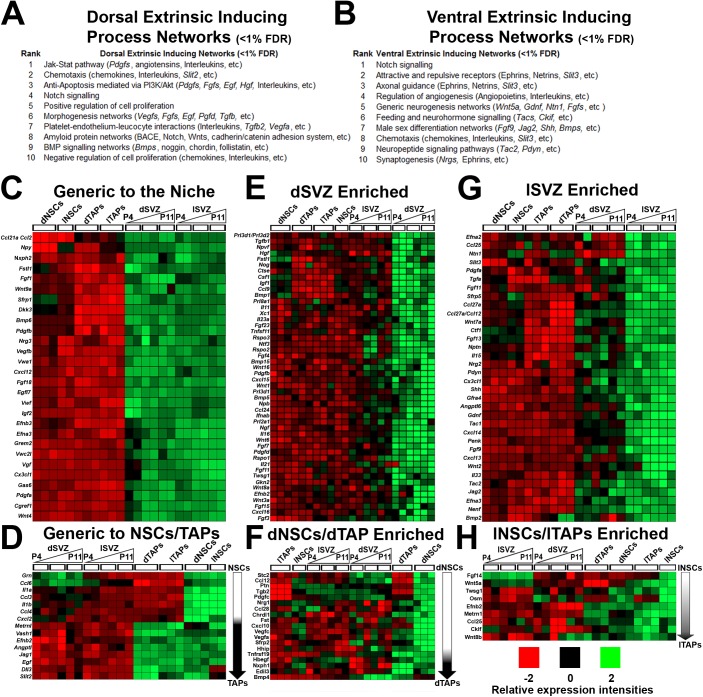
Spatial expression profiles of secreted signaling factors in subventricular zone (SVZ) microdomains. **(A, B)** Significantly filtered genes in each region-derived sample were analyzed on GeneGo Metacore for process networks and ranked numerically according to their false discovery rate (FDR) significance (<1%). NB: process networks categories were shortened to fit. (**C, D)** Heatmaps of genes generic and stable within microdomains across the varied time points are plotted, including those common in isolated neural stem cells (NSCs)/transient amplifying progenitors (TAPs) **(D)**. (**E-G)** Heatmaps of genes enriched in region-specific microdomains (generally stable temporal expression) versus the adjacent microdomain and NSCs/TAPs. The same was performed for region specific NSCs/TAPs **(F, H)**. Note: the overlapping expression profiles for region-specific NSCs with TAPs. dNSCs, dorsal NSCs; dTAPs, dorsal TAPs; dSVZ, dorsal SVZ; lNSCs, lateral NSCs; lTAPs, lateral TAPs; lSVZ, lateral SVZ.

The SVZ contains NSCs and their progeny, the transient amplifying progenitors (TAPs), which generate both NPs and OPs. The SVZ can be subdivided into discrete spatial microdomains (or niches) from which distinct neural lineages originate. While subtypes of GABAergic interneurons originate from all SVZ regions, the dorsal SVZ (dSVZ) additionally gives rise to glutamatergic NPs and is the primary source of forebrain OPs (reviewed in [6,7,13]). To identify the molecular hallmarks that determine cell fate within these microdomains, we previously generated whole transcriptome datasets of NSCs, TAPs, and their respective SVZ niches at postnatal day (P)4, P8, and P11 [1], which correspond to the postnatal period of greatest germinal activity and lineage diversity [6,14]. Here, we interrogated these datasets to identify signaling and metabolic processes that are unique to NSCs, TAPs, and their respective SVZ niches. Transcripts enriched in dorsal versus lateral datasets were compared using GeneGO Metacore for Process Networks, and the function of individual genes were classified using http://www.genecards.org. The top ten Metacore categories in each microdomain were ranked (Fig 1A and 1B), and only two categories overlapped, namely “Chemotaxis” and “Notch signaling,” stressing the importance of these pathways within the neurogenic niche as well as highlighting the existence of discrete signaling processes that are specific to the dorsal and lateral SVZ microdomains (Fig 1A and 1B). Among enriched transcripts generic to the SVZ niche, those coding for secreted signaling factors (grouped here for simplicity as “morphogens”) were prominent in both SVZ microdomains (Fig 1C); many of these were enriched in NSCs/TAPs (Fig 1D). Examination of the dorsal and lateral SVZ revealed a large number of genes differentially enriched within the two microdomains (Fig 1E–1H). Notably, the dSVZ contained the greatest number of genes that were uniquely expressed (Fig 1E), in line with the greater diversity of lineages originating from this microdomain. In particular, the Wnt ligands were specific to the dSVZ, whilst Shh was specific to the lateral SVZ, in accordance with evidence that these signaling pathways have key roles in dorsalization and ventralization of the SVZ, respectively [12,15]. In addition, several members of the TGFβ/Bmp family and their pathway inhibitor *Noggin* were enriched in the dSVZ (Fig 1E), indicating they may have a specific role in driving cell fate in this microdomain. The lateral SVZ was enriched in the proneural determinants *Bmp2* and *Tgfa* [16], as well as an abundance of chemokines and secreted molecules with undefined roles in neurogenesis (Fig 1G and 1H). A number of *Fgf* ligands were specific to the dorsal or lateral SVZ, indicative of functional divergence of FGF signaling within the microdomains (Fig 1E and 1G). Together, these results highlight major regionalization of signaling pathways within the SVZ, supporting the possibility they could be directly targeted to instruct lineage commitment of NSCs.

### SPIED/CMAP identification of small molecules for manipulating SVZ regionalization and NSC fate

**Fig 2:**
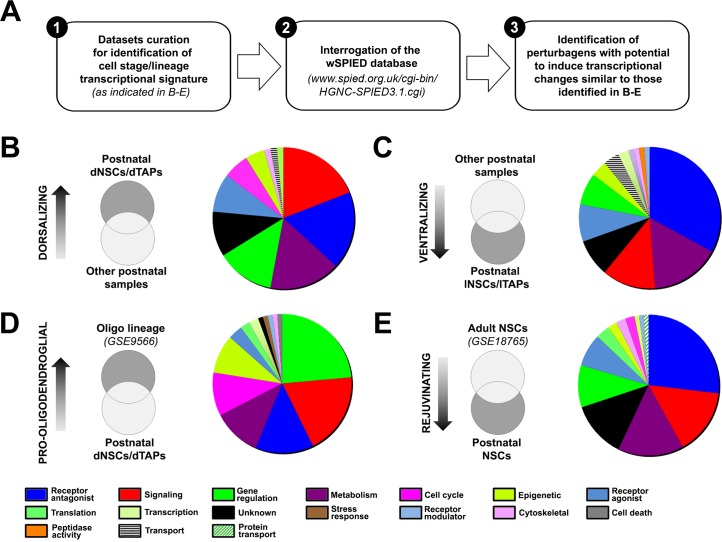
Connectivity map (CMAP) identification of perturbagens that promote region-specific subventricular zone (SVZ) signatures, oligodendrogenesis, and reactivation of adult neural stem cells (NSCs). **(A)** Schematic representation of the experimental flow. Microarray datasets were compared for obtaining expression signatures (detailed in Materials and Methods) and uploaded onto searchable platform-independent expression database (SPIED) to interrogate the CMAP for obtaining a list of perturbagens. These were further inspected for their known protein targets, categorized and presented in the figure as pie charts (ordered clockwise following their ranking order). Note that small molecules related to distinct top-ranked categories are observed in the different analysis. Genes enriched in **(B)** dNSCs/dTAPs compared to other early postnatal datasets (i.e., dorsalization); **(C)** lNSCs/lTAPs compared to other early postnatal datasets (i.e., ventralization); **(D)** oligodendrocyte (OL) lineage cells compared to dNSCs and dTAPs; **(E)** Postnatal NSCs compared to adult NSCs. dNSCs, dorsal NSCs; dTAPs, dorsal TAPs; lNSCs, lateral NSCs; lTAPs, lateral TAPs.

We applied a novel pharmacogenomics approach to probe SVZ regionalization. To this end, a meta-analysis was performed to identify relationships between the transcriptional signatures of SVZ niches and/or lineages to those induced by exposure to small bioactive molecules in different contexts. This CMAP approach allows the identification of small bioactive molecules capable of inducing transcriptional changes similar to those observed in the queried samples and therefore to potentially manipulate cell fate in the SVZ (Fig 2A) [3,4,17]. Small molecules were ranked according to the number of genes that are altered, referred to as “target genes” (See Table 1A–1D), and the protein targets of each small molecule were classified according to Gene Ontology (GO) terms (Fig 2B–2E; see Materials and Methods for further details).

**Table 1:** Top-ranked small molecules identified from SPIED/CMAP analysis of small molecules that promote (A) dorsalization of the SVZ, (B) ventralization of the SVZ, (C) oligodendrogenesis, and (D) neurogenesis. Small molecules are ranked according to the largest numbers of “target” or “perturbed” genes. For the full list, please refer to corresponding S1–S4 Tables.

**(A) DORSALIZATION**		**(B) VENTRALIZATION**	
**Small molecules**	**Number of target genes**	**Small molecules**	**Numbers of target genes**
GW-8510	1367	verteporfin	735
ciclopirox	475	cephaeline	598
AR-A014418	456	terfenadine	452
oxprenolol	366	prenylamine	420
streptomycin	357	3-nitropropionic_acid	396
cinchonine	353	abamectin	361
alfaxalone	323	rescinnamine	354
caffeic_acid	323	etanidazole	336
oxytetracycline	323	dicycloverine	284
azaperone	301	5707885	263
**(C) OLIGODENDROGENESIS**		**(D) REJUVENATION**	
**Small molecules**	**Number of target genes**	**Small molecules**	**Numbers of target genes**
trichostatin_A	3292	adiphenine	1227
tanespimycin	2732	helveticoside	1157
**LY-294002**	**2296**	thapsigargin	1070
vorinostat	2247	**AR-A014418**	**998**
GW-8510	2093	anisomycin	989
camptothecin	1927	lanatoside_C	865
phenoxybenzamine	1871	viomycin	776
H-7	1803	monensin	762
sirolimus	1783	nadolol	761
irinotecan	1752	podophyllotoxin	757

CMAP, connectivity map; SPIED, searchable platform-independent expression database; SVZ, subventricular zone.

Small molecules that may drive NSC/TAP dorsalization or ventralization were identified by comparing the transcriptome of dorsal and lateral NSCs/TAPs (S1 and S2 Tables), as summarized in Table 1A and 1B. Significantly, the GSK3β inhibitor AR-A014418, which we have previously demonstrated dorsalizes the SVZ [10], was identified in the top ten dorsalizing pertubagens (Table 1A). Conversely, 3-nitropropionic acid ranked highly in the ventralizing screen (Table 1B) and is an activator of GSK3β [18], which we have shown represses SVZ dorsalization [15]. Drugs targeting the ventralizing Shh signaling pathway [12] were also identified, such as tolnaftate, which has been described as inhibiting Shh signaling [19]. Altogether, these findings help validate our approach (see below). Among other small molecules, the most prominent category was “Receptor antagonists” (Fig 2B and 2C), consistent with neurotransmitters being major regulators of neurogenesis in the SVZ [20]. Notably, ventralizing perturbagens targeting muscarinic acetylcholine (mACh) receptors were highly ranked, e.g., terfenadine (Table 1B), suggesting mACh receptors are important determinants of interneuron specification in the lateral SVZ [21]. The two other prominent categories of small molecules were associated with “Signaling” and “Metabolism” (Fig 2B and 2C), which included the most highly ranked dorsalizing perturbagen GW-8510, a potent Cdk inhibitor (Table 1A), and ciclopirox, an inhibitor of prolyl-4-hydroxylase that promotes Notch signaling and NSC activation [22,23]. The most highly ranked ventralizing perturbagen was verteporfin (Table 1B), which alters downstream transcriptional activity of the hippo pathway to regulate cell cycle and neuronal differentiation [24].

Small molecules that may promote oligodendrogenesis were identified by comparing the transcriptome of dorsal NSCs/TAPs (i.e., the main postnatal forebrain source of OLs) with publicly available transcriptional datasets of oligodendroglial lineage cells [25]. The results are presented in S3 Table and summarized in Table 1C. Most small molecules were related to “Gene regulation” and”Signaling” (Fig 2D). Many from the latter category, including two of the top ten ranked pro-oligodendrogenesis drugs LY-294002 and sirolimus, are inhibitors of PI3K/Akt/mTor signaling (Table 1C), which acts downstream of several ligands enriched in the dSVZ (Fig 1E). Notably, many of the small molecules associated with oligodendrogenesis were within “Epigenetic” and “Cell cycle” categories, which barely featured in the other SPIED analyses (Fig 2B–2E). The highest ranking amongst these were trichostatin-A and vorinostat (Table 1C), which are potent inhibitors of histone deacetylases (HDACs) with broad epigenetic activities, consistent with evidence that HDACs repression is required during NSC differentiation into OPs [26,27]. Similarly, the higher ranking of the Cdk inhibitors GW-8510 and camptothecin (Table 1C) highlights the importance of inhibition of Cdks in regulating cell cycle progression and differentiation of OPs from NSCs, in support of previous studies [28].

Small molecules that may rejuvenate the adult SVZ were identified by comparing the transcriptome of postnatal NSCs [1] and adult NSCs [29] (Fig 2E; S4 Table). The aim of this approach was to identify key transcriptional changes underlying the decline in the activity and loss of competence of NSCs that occurs in adulthood, which is attributed to the combined up-regulation of inhibitory and down-regulation of positive cues [30], and is critical for the response of NSCs to brain injury and degeneration [31,32]. In this manner, key small molecules were identified for “rejuvenating” adult NSC (Fig 2E; Table 1D), approximately 20% of which overlapped with those required in dorsalizing or ventralizing the postnatal SVZ, and were categorized as “Receptor antagonist,” “Signaling,” and “Metabolism.” Some interesting candidates included antagonists for α/β adrenergic receptors, including the highly ranked nadolol (Table 1D), which have been described to promote NSC activation from quiescence in the dentate gyrus [33] and enhance NP survival following exit from the SVZ [34]. A key signaling pertubagen was monensin, which impedes TGFβ processing [35], a major neurogenesis inhibitory factor during aging [36]. Significantly, the GSK3β inhibitor AR-A014418 was one of the top ranking “rejuvenating” small molecules (see below), which we have shown promotes the genesis of glutamatergic NPs in the postnatal SVZ via the canonical Wnt signaling pathway [10,15].

Based on these SPIED/CMAP analyses, LY-294002 and AR-A014418 were identified as promising agents that may specifically regulate oligodendrogenesis (Table 1C) or neurogenesis (Table 1D), respectively, and were selected for further analysis to resolve signaling-to-transcriptional interactions by Genego Metacore network visualization and in vivo validation.

### LY-294002 induces transcriptional changes that promote oligodendrogenesis

**Fig 3:**
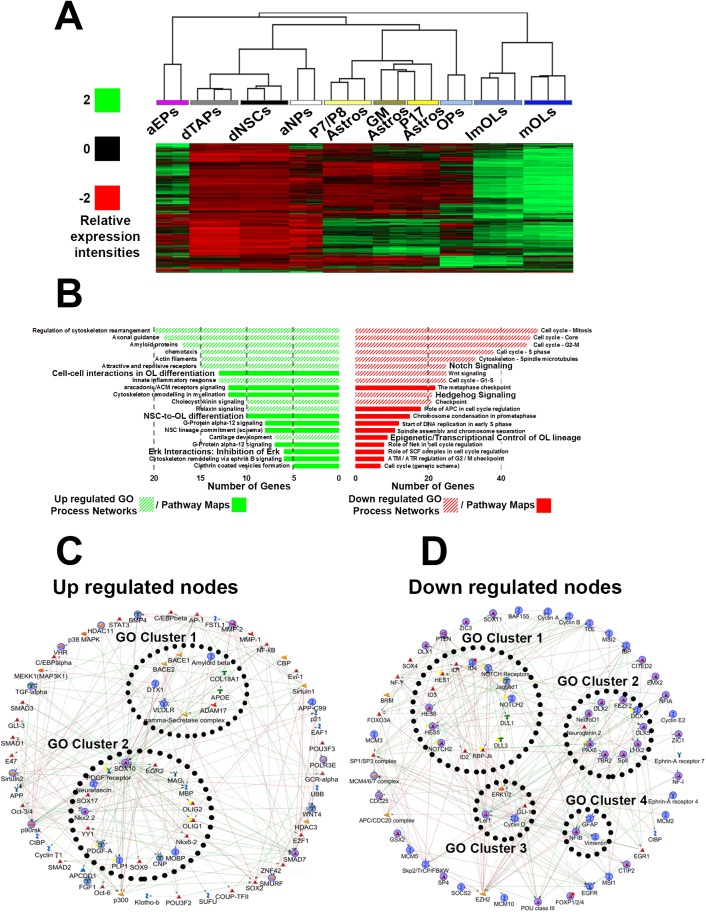
Connectivity map (CMAP) pathway and network analysis for LY-294002 target genes. Gene list generated to obtain the drug profiles for LY-294002 (S5 Table) was utilized to compile the CMAP “LY-294002 target-gene” list, and genes prospectively up-regulated are presented as a heatmap in **(A)** showing enrichment in later oligodendrocyte (OL) lineage cells and down-regulation in dorsal neural stem cells (NSCs) or transient amplifying progenitors (TAPs). aEPs, adult ependymas; aNPs, adult neuronal precursors (NPs); GM, grey matter; astros, astrocytes; imOLs, immature OLs; mOLs, mature/myelinating OLs. **(B)** Prospectively up-regulated or down-regulated genes analyzed by Genego Metacore for GO Pathway Maps and Process Networks, and lists are ranked according to significance (false discovery rate [FDR] <2%)/numbers of genes present in each of the categories. **(C, D)** Short path network to visualize highly connected signaling-to-transcriptional nodes were performed for up- and down-regulated by LY-294002 target genes. Internal clusters were grouped by selecting the “link GO objects” in GeneGo Metacore. Highlighted blue objects are directly within data and the remaining are within the background (or basal) data.

**Fig 4:**
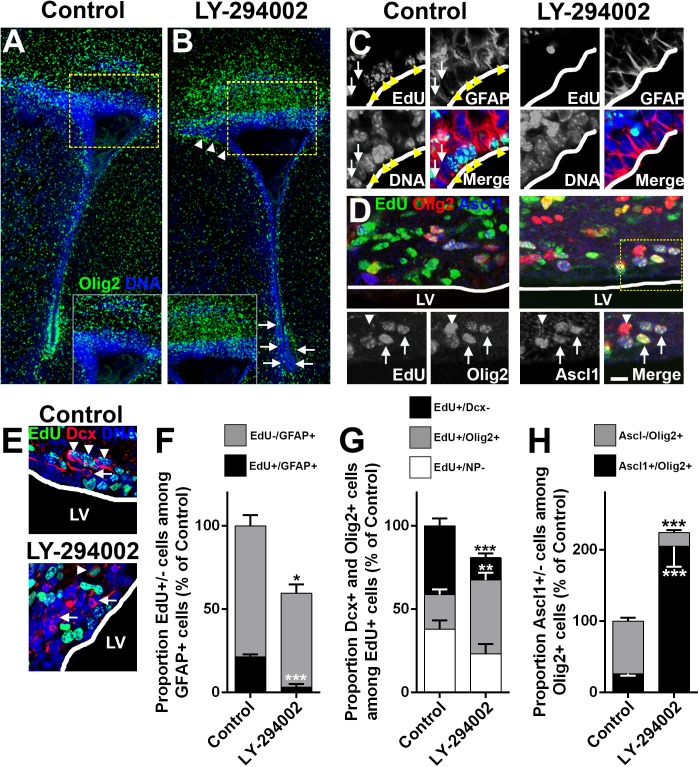
LY-294002 promotes dorsal subventricular zone (dSVZ)-oligodendrogenesis. Pups were treated for 3 days with LY-294002 or saline/DMSO and examined by immunolabeling. **(A, B)** Periventricular sections show greater Olig2 immunostaining in LY-294002 in more dorsal periventricular regions compared to controls, illustrated in expanded insets. In the lateral SVZ, LY-294002 reduced Olig2 expression, as indicated by arrows **(B)**. Arrowheads in **(B)** show reduced nuclei density in the dorsolateral horn of the SVZ where neuronal precursors (NPs) migrate. Scale bar in **(A)** = 200 μm. **(C)** Arrowheads show a loss of EdU in GFAP+ cells directly facing the lateral wall in LY-294002 compared to controls. Arrows show examples GFAP+ cells that have not incorporated EdU that were increased following LY-294002. **(D)** Single plane confocal micrographs show greater EdU+\Olig2 colocalization in LY-294002 and single panel captions of single planes illustrate that most newly generated Olig2+ cells co-express EdU and Ascl1 (arrows) or have absent or lower levels of Ascl1 (arrowheads). Scale bar in **(D)** = 10 μm in captions of **(D)**, 15 μm in main panels of **(D)**, 15 μm in **(C)**, and 10 μm in **(D)**. **(E)** Confocal micrographs illustrate a lower density of Dcx+ cells in LY-294002 and a loss of their proliferative status (compare Dcx+ cells with arrowheads to those marked by arrows). **(F-H)** Quantification of changes in GFAP+/EDU+ or EdU- cells directly facing the wall of the lateral ventricle **(F).** Quantification of changes in EdU+ cells expressing progenitor markers (Dcx, Olig2, or none of these markers [NP-]) **(G)**. Quantification of changes in Olig2+/Ascl1+ or Ascl1- cells **(H)**. Data are mean ± standard error of the mean (SEM) normalized to controls (*n* = 5 for control and LY-294004 for all quantifications); significance was tested using unpaired *t* test throughout versus respective control; **p < 0.01; ***p < 0.001.

LY-294002 is a widely used and highly specific inhibitor of PI3K/Akt. Expression of the target genes of LY-294002 in SVZ cell types/lineages (S5 Table) was compared by hierarchical clustering, highlighting their association with late-stage OLs compared to other cell lineages, including NSCs/TAPS of the dSVZ (Fig 3A). Further target genes analysis provided additional information on the mode of action and predicted effects of LY-294002. Categorizing target genes for GO Pathway Maps and Process Networks revealed up-regulation of genes associated with oligodendrogenesis and myelination and down-regulation of genes related to cell cycle behavior and neurogenesis (Fig 3B). Finally, Genego Metacore network visualization was applied to resolve signaling-to-transcriptional interactions (detailed in Materials and Methods). LY-294002 up-regulated transcriptional nodes were associated with oligodendrogenesis (Fig 3C), while down-regulated nodes included Notch signaling, proneuronal TFs, and astroglial-related genes (Fig 3D). Altogether, LY-294002 appeared as a strong candidate for inducing specifically oligodendrogenesis in the postnatal SVZ. It was infused into the CSF of the lateral ventricle, commencing at P8, and the effects on the SVZ were determined at P11 by immunostaining (Fig 4) and biochemical and quantitative PCR (qPCR) analysis of its target genes (S1A and S1B Fig), as described in our previous studies [10,15]. Intraventricular infusion to achieve a CSF concentration of 3 μM LY-294002 effectively inhibited Akt phosphorylation, the immediate target of PI3K, throughout the SVZ (S1A Fig) and rapidly and specifically promoted oligodendrogenesis in the dSVZ (Fig 4A and 4B). Quantification performed through the rostro-caudal axis of the lateral ventricle revealed a pronounced induction of the OL lineage marker Olig2, particularly in the most dSVZ region (Fig 4A and 4B). NSCs were identified as glial fibrillary acidic protein (GFAP) immunopositive cells in direct contact with the lateral ventricle wall, and their proliferative state was assessed using 5-ethynyl-2 deoxyuridine (EdU; mice received a single intraperitoneal [i.p.] injection of EdU at P8). Compared to controls, GFAP immunoreactivity and the extent of GFAP+/EdU colocalization were significantly reduced following LY-294002 infusion (Fig 4C and 4F), indicating a general loss of both proliferative and nonproliferative GFAP+ NSCs, consistent with evidence of their precocious differentiation into OPs. Immunolabeling for Dcx and Olig2 in combination with EdU to identify NPs and OPs, respectively, revealed different effects of LY-294002 on these two lineages. Notably, the numbers of Olig2+/EdU+ OPs were increased dramatically (Fig 4D and 4G), and a greater proportion of these cells expressed Ascl1 (Fig 4D and 4H), whereas Dcx+/EdU+ NPs were reduced, as was the overall number of Dcx+ cells in the dSVZ (Fig 4E and 4G). This indicates that LY-294002 promoted the early stages of oligodendrogenesis at the expense of neurogenesis. In addition, OL differentiation was also enhanced as revealed by a doubling of proteolipid protein (PLP)-DsRed+ OLs and a subsequent 30% enhancement in myelination as revealed by myelin index measurements (S1C Fig) by the end of the treatment.

Importantly, qPCR of the microdissected SVZ (S1B Fig) confirmed LY-294002 acts via the target genes/nodes identified by the target gene analysis (Fig 3) and provided additional information on its modes of action. Analysis revealed *Fgf2* and *Igf1* were not increased (S1B Fig), indicating they were not the mechanism of action of LY-294002. Conversely, LY-294002 significantly decreased Shh signaling, which promotes SVZ ventralization, together with Notch signaling (S1B Fig), which stimulates NSCs self-renewal and is a major rate-limiting determinant of OL differentiation [37,38]. Overall, these analyses support that LY-294002-mediated PI3K/Akt inhibition promotes an environment permissive to oligodendrogenesis while inhibiting signaling pathways that promote neuronal cell fates.

### AR-A014418 induces transcriptional changes that promote rejuvenation of the adult SVZ

**Fig 5:**
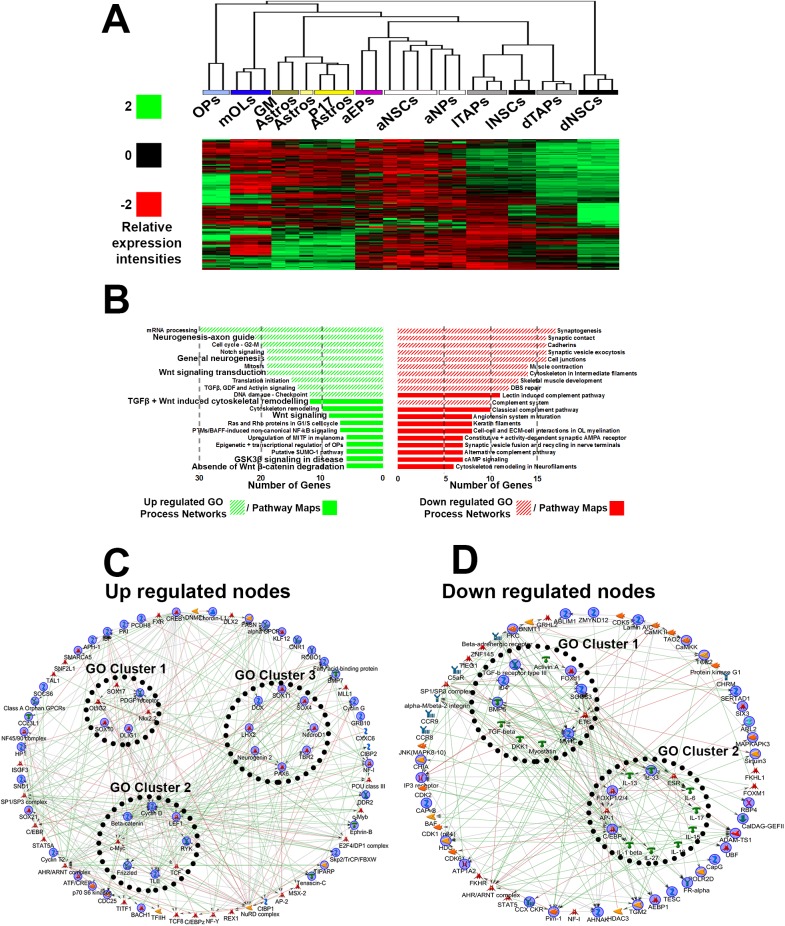
Connectivity map (CMAP) pathway and network analysis for AR-A014418 target genes. Gene list generated to obtain the drug profiles for AR-A014418 (S6 Table) was utilized to compile the CMAP “AR-A014418” target-gene list, and genes prospectively up-regulated are presented as a heatmap in **(A)** showing enrichment in earlier postnatal dorsal subventricular zone (dSVZ) cells. aEPs, adult ependymas; aNPs, adult neuronal precursors; GM, grey matter; astros, astrocytes; mOLs, mature/myelinating oligodendrocytes; aNSCs, adult neural stem cells (NSCs); dNSCs, dorsal NSCs; dTAPs, dorsal transient amplifying progenitors (TAPs); lNSCs, lateral NSCs; lTAPs, lateral TAPs. **(B)** Prospectively up-regulated or down-regulated genes analyzed by Genego Metacore for GO Pathway Maps and Process Networks, and lists are ranked according to significance (false discovery rate [FDR] <1%)/numbers of genes present in each of the categories. **(C, D)** Short path network to visualize highly connected signaling-to-transcriptional nodes were performed for up- and down-regulated ARA-014418 target genes. Internal clusters were grouped by selecting the “link GO objects” in GeneGo Metacore. Highlighted blue objects are directly within data and remaining objects are within the background (or basal) data.

AR-A014418 is a highly specific inhibitor of GSK3β, which the SPIED/CMAP analysis identified as having one of the highest number of target genes associated with rejuvenation (Table 1D) as well as being positively related to dorsalization of the SVZ (Table 1A) and negatively related to ventralization (Table 1B). Consistent with this, the target genes of AR-A014418 were enriched more prominently in dorsal NSCs/TAPs and OPs, than other cell types (Fig 5A). Furthermore, pathway/network analysis of AR-A014418 target genes revealed an up-regulation of multiple categories related to neurogenesis, oligodendrogenesis, and Wnt signaling (Fig 5B), consistent with our recent evidence that AR-A014418 promotes generation of glutamatergic NPs and OPs from the postnatal dSVZ via canonical Wnt signaling [10,15]. Genego Metacore network visualization identified up-regulated nodes mainly consisting of transcriptional regulators, notably TFs associated with neurogenesis (Fig 5C; e.g., *Tbr2*, *NeuroD1*, *Pax6*) and oligodendrogenesis (Fig 5C; e.g., *Olig1/2*, *Sox10*). Down-regulated nodes included members of pro-inflammatory cytokines (Fig 5D), such as *IL-33*, that likely inhibit neurogenesis [39], as well as *Id4*, which is up-regulated in adulthood and is a potent inhibitor of neurogenesis [40]. These analyses support AR-A014418 as a strong candidate for promoting lineage and signaling pathways that are characteristic of the early postnatal SVZ, whilst down-regulating inhibitory factors associated with the decline in neurogenic capacity in the adult.

**Fig 6:**
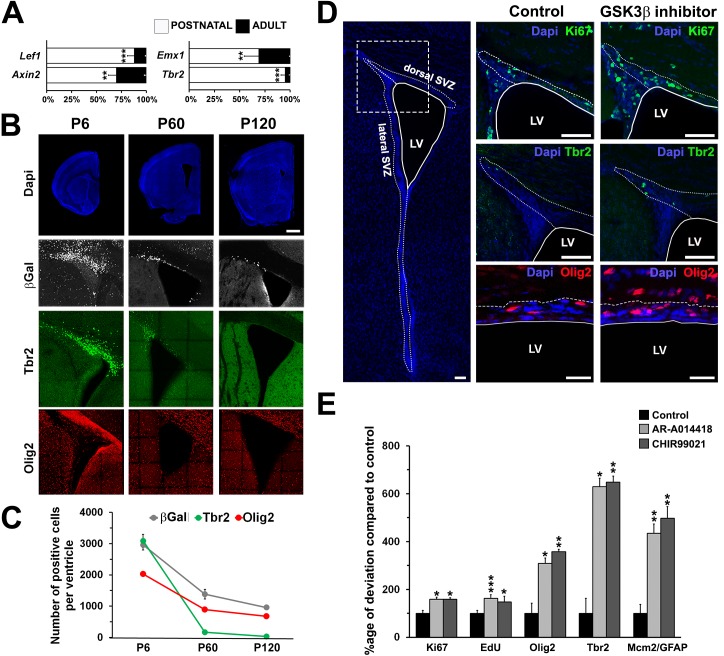
Pharmacological stimulation of Wnt/β-catenin signaling rescues oligodendrocyte precursor (OP) and glutamatergic neuron progenitor numbers in the adult mouse. **(A)** Quantitative PCR (qPCR) analysis reveals a pronounced decrease of Wnt targets genes *Lef1* and *Axin2* and pallial *Emx1* and *Tbr2* transcripts expression in the dorsal subventricular zone (dSVZ) between P6 and P60 (*n* = 3 for P6 and P60). Results are expressed as a percentage and normalized in comparison with *Gapdh* level of expression and compared using unpaired *t* test. **(B)** Representative coronal sections illustrating the pronounced and rapid decrease of Wnt canonical signaling in the βGal reporter mouse (βGAL+) and the parallel decrease of glutamatergic NPs (Tbr2+) and OPs (Olig2+) in the SVZ of mouse brain at the age of 6 d (P6), 2 mo (P60), and 4 mo (P120) (*n* = 3 individual animals for each time point). **(C)** Quantification of the average number of βGAL+, Tbr2+, and Olig2+ cells in the dorsal wall of the SVZ in P6, P60, and P120 mice (3 animals per age). **(D)** Representative pictures of Ki67, Tbr2, and Olig2 expression in the adult (P90) SVZ before and after treatment with GSK3β inhibitors (AR-A014418, not shown, and CHIR99021, shown). **(E)** Percentage increase of proliferation (Ki67, EdU), OPs (Olig2), glutamatergic NPs (Tbr2), and NSC (Mcm2/GFAP) numbers following intraventricular infusion of AR-A014418 (3–10 μM) and CHIR99021 (3–10 μM). Values are normalized compared to the controls (*n* = 5 for each of control, AR-A014418, and CHIR99021). Error bars represent standard error of the mean (SEM). ***, p < 0.001; **, p < 0.01; *, p < 0.05; *t* test. Scale Bar = 1 mm **(B)** and 50 μm **(D)**.

An age-related decline in SVZ activity was confirmed by qPCR analysis of adult SVZ microdomains, which indicated a parallel decline in neurogenic potential and canonical Wnt/β-catenin signaling in the dSVZ (S2 Fig). This was confirmed by an observed sharp decline in the expression of *Lef1* and *Axin2* and the dSVZ\glutamatergic NP markers *Emx1* and *Tbr2* (Fig 6A). Analysis of BAT-gal mice [41] further demonstrated a decline in Wnt/β-catenin activity between P6 and P60, together with a decrease in the densities of Tbr2+ NPs and to a lesser extent Olig2+ OPs in the adult and their apparent loss by P120 (Fig 6B and 6C). These analyses reveal that the neurogenic capacity and lineage diversity of the dSVZ declines in the adult brain [30,42] and, based on the SPIED/CMAP analysis, we predicted GSK3β inhibitors are strong candidates for rejuvenating the adult dSVZ.

The effects of the GSK3β inhibitors AR-A014418 and second generation inhibitor CHIR99021 on dorsal lineages in the adult SVZ were examined directly in vivo by infusion into the CSF of the lateral ventricle (Fig 6D and 6E and S3–S5 Figs). First, these agents were tested in postnatal mice to confirm our previous findings that their infusion into the CSF effectively inhibits GSK3β activity and stimulates Wnt/β-catenin signaling in the dSVZ (S3 Fig) [15] and promote the generation of glutamatergic NPs and OPs (S4 Fig). Next, we examined the effects of AR-A014418 or CHIR99021 in the adult (P90) dSVZ; the agents were infusion into the lateral ventricle caudally to ensure no damage to the SVZ by the injection procedure, whilst ensuring effective distribution of agents at a concentration of 3–10 μM at the SVZ, which is rostral to the injection site (S5 Fig). Treatment with AR-A014418 and CHIR99021 dramatically stimulated the germinal activity of the adult dSVZ, increasing proliferation as revealed by Ki67 immunolabeling and EdU incorporation, with profound effects on Mcm2/GFAP+ NSCs and Tbr2+ glutamatergic NPs, which were respectively increased 5-fold and 6-fold (Fig 6D and 6E). There was also an increase in oligodendrogenesis, as evinced by a 3-fold increase in the number of Olig2+ cells (Fig 6D and 6E). Importantly, careful analysis revealed no marker co-expression (i.e., Olig2/Tbr2 and Dlx2/Tbr2, <50 cells/brain in five animals), supporting appropriate and lineage-specific progenitor specification. Thus, infusion of GSK3β inhibitors was able to rejuvenate the SVZ by promoting the reemergence of lineages associated with early postnatal life, as predicted by the SPIED/CMAP analysis.

### Administration of small bioactive molecules that promotes SVZ germinal activity in a model of premature brain injury

**Fig 7:**
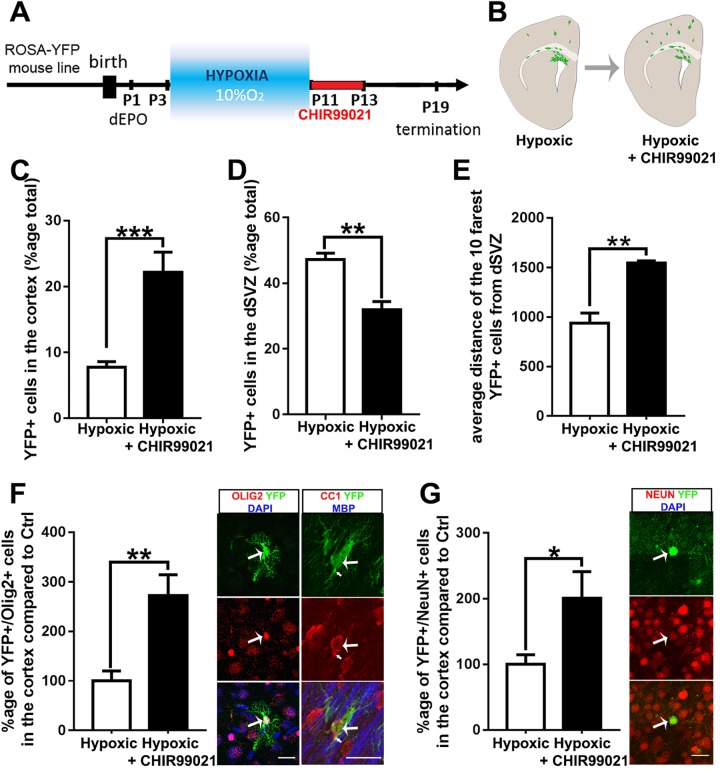
CHIR99021 promotes dorsal subventricular zone (dSVZ)-derived cortical oligodendrocyte (OL) regeneration following chronic hypoxia. **(A)** Schematic representation of the experimental workflow. A Cre plasmid was electroporated in the dSVZ of ROSA-YFP mice 1 day after birth (P1) for permanent labeling of dorsal neural stem cells (NSCs). Mice were placed in a hypoxic chamber containing 10% O_2_ from P3 to P11 then subjected to intranasal CHIR99021 administration from P11 to P13. Animals were sacrificed at P19 for analysis of recombined cell number, migration, and differentiation. **(B)** Schematic representation of the results quantified in **(C-E)**. **(C-D)** CHIR99021 treatment following hypoxia leads to a decrease of YFP+ cells in the dSVZ **(C)**, paralleled by a concomitant increase in the cortex (*n* = 4 for hypoxic and *n* = 4 for CHIR99021) **(D)**. **(E)** The average distance of the ten farthest YFP+ cells from the dorsal SVZ is increased following CHIR99021 treatment (*n* = 4 for hypoxic and *n* = 3 for CHIR99021). **(F-G)** CHIR99021 treatment promote de novo oligodendrogenesis **(F)**, YFP+/Olig2+, and neurogenesis **(G)**, YFP+/NeuN+; left confocal micrographs following hypoxia (*n* = 7 for hypoxic and *n* = 6 for CHIR99021). Right confocal micrographs show expression of CC1 in YFP+ cells in the most superficial cortical layers, supporting the successful differentiation of the newborn OLs (large arrow) that support myelin (small arrow shows colocalizing YFP+/MBP+ myelinated fibers) in CHIR99021 treated animals. ***p < 0.001; **, p < 0.01; *, p < 0.05; *t* test used throughout. Scale bars = 20 μm throughout.

The results reported above validate the SPIED/CMAP-based approach for lineage-specific manipulation of SVZ germinal activity at various ages. Next, we explored the regenerative potential in a neuropathological context in a model of premature injury that leads to diffuse oligodendroglial and neuronal loss throughout the cortex [43]. To investigate the potential of small bioactive molecules to promote the spontaneous cellular regeneration previously observed in this model [44,45], we selected the GSK3β inhibitor CHIR99021 for its predicted capacity to induce dorsal lineages, i.e., oligodendrogenesis and neurogenesis [10] (Fig 2), and high activity at low concentrations in inducing dorsal lineages (S4C Fig). Dorsal electroporation of a Cre plasmid in Rosa-YFP Cre reporter mice allowed long-term labeling and fate mapping of dorsal NSCs. Intranasal CHIR99021 delivery in hypoxic animals led to a significant decrease in the number of YFP+ cells in the dSVZ, while their number concomitantly increased in the cortex (Fig 7C and 7D). This efficient cortical cellular recruitment was accompanied by a significant enhancement of migration of YFP+ cells following CHIR99021 treatment (Fig 7B and 7E). Phenotypic characterization of the cells revealed significantly enhanced oligodendrogenesis (YFP+/Olig2+, Fig 7F), regeneration of new myelinating OLs (YFP+/CC1+/MBP+, Fig 7F), as well as increased neurogenesis (YFP+/NeuN+, Fig 7G) following hypoxia and CHIR99021 treatment. Expression of postmitotic markers CC1 and NeuN in OLs and neurons, respectively, support their successful differentiation following CHIR99021 treatment (Fig 7G and 7H). These results illustrate the capacity of small bioactive molecules identified in our bioinformatic approach to promote regeneration following forebrain injury.

## Discussion

Controlling the fate of NSCs to generate neurons and OLs would represent a key therapeutic strategy in neuroregenerative medicine. In the present study, we used a novel SPIED/CMAP strategy to identify small molecules that are predicted to regulate transcriptional changes associated with neurogenesis in the SVZ neurogenic niche. Importantly, we validate this approach by demonstrating that two of the identified small molecules, which target PI3K/Akt and GSK3β, were able to differentially direct the fate of NSCs in vivo, to promote oligodendrogenesis and neurogenesis, in the postnatal and adult SVZ. Moreover, we show that this approach can be used to promote regeneration in a clinically relevant mouse model of postnatal brain hypoxia, which demonstrates the power of the SPIED/CMAP-based approach for repurposing of clinically approved small molecules for promoting regeneration in neurodegenerative and demyelinating diseases.

Transcriptome profiling revealed for the first time the marked heterogeneity of secreted ligands within the dorsal and lateral microdomains of the postnatal SVZ that are likely to contribute towards the regionalized fate of NSCs by regulating downstream transcriptional programs [1,10,12]. These regional differences suggested to us that specific signaling pathways could be probed as a means to manipulate SVZ regionalization as well as the lineages associated with specific SVZ microdomains. To this end, we provide the first interrogation of the SVZ using the CMAP initiative, which has been used extensively in other systems (reviewed in [4]). We identified small molecules that have the potential to manipulate region and/or lineage-specific gene expression signatures. Small molecules were further prioritized by additional analysis of their target genes. First, small molecules can be prioritized by the number of “target genes” (Table 1). Additionally, “target genes” were examined by further by GO analysis, which enabled us to prioritize small molecules predicted to act on select lineages/signaling pathways and exclude those with undesirable side effects (e.g., cell death). Applying these selection criteria, we selected LY-294002 and AR-A014418 for validation in vivo and showed that they stimulate the genesis of specific lineages during development and in adulthood.

To target oligodendrogenesis, we selected the specific PI3K inhibitor LY-294002 on the basis that our SPIED/CMAP analyses predicted an up-regulation of signatures relevant to OL lineage cells. Network analysis revealed that dorsal-derived lineages are promoted by via PI3K/Akt signaling, which was highly ranked in analysis performed in Fig 1A, implying novel fate-specification roles. In addition, in vitro studies indicate an important role for PI3K/Akt signaling in OL development [46,47]. Our findings demonstrate for the first time that intraventricular infusion of LY-294002 specifically targeted oligodendrogenesis in vivo in the dSVZ, which is the primary source of forebrain OLs in the postnatal brain [8]. A further novel finding was that Notch2-signaling is a target of PI3K/Akt in vivo and may be a primary mechanism regulating fate decision of NSC along an oligodendroglial path [37,38], which had not been predicted previously from genetic or in vitro studies. It is significant, therefore, that ciclopirox, an inhibitor of prolyl-4-hydroxylase that promotes Notch signaling and NSC activation [22,23], was the second most highly ranked pertubagen predicted to promote oligodendrogenesis. Other factors identified by our analyses, and worthy of further examination, inhibit HDACs, which is essential for generation of OPs [26]. Indeed, our analyses indicate the key to promoting oligodendrogenesis in the SVZ is to inhibit signaling pathways that promote neuronal cell fate, and future strategies should examine the therapeutic potential of combinations of small molecules.

Gene expression profiling of the SVZ demonstrated a marked decline in NSC activity during adulthood, in support of other studies indicating an age-related loss of specific signaling cues that promote NSC proliferation and fate commitment [42,48,49]. Most notable was the decline in canonical Wnt/β-catenin activity, which is consistent with an increase in Wnt inhibitors during adulthood [50]. It is significant, therefore, that our study demonstrates that small molecule inhibitors of GSK3β identified by the SPIED/CMAP analysis act as mimetics of Wnt/β-catenin and successfully reverse the decline in specific neural lineages in the adult SVZ. Significantly, we identified that the GSK3β inhibitors AR-A014418 and CHIR99021 have a profound effect on germinal activity in the dSVZ of 3-mo-old mice. This was primarily illustrated by the pronounced effect on glutamatergic NPs, although OPs were also increased.

In summary, our analyses provide a comprehensive catalogue of small molecules that can be used to manipulate SVZ microdomain-specific lineages. The power of this approach is highlighted by our results, demonstrating that two of these compounds, LY-294002 and AR-A014418, stimulate oligodendrogenesis and glutamatergic NPs generation in postnatal and adult contexts. Moreover, experiments performed in a model of postnatal brain injury reveal that this strategy can be applied to promote SVZ germinal activity in a regenerative context. Although further work is required to fully characterize the functional outcome of this repair, these encouraging results will stimulate further studies on repurposing other small molecules and determine their potential for lineage-specific manipulation of SVZ germinal activity and regeneration in multiple neuropathologies. In this regard, it is noteworthy that identified small molecules, for example GSK3β inhibitors, may be useful for reactivating Wnt-signaling, which is down-regulated during impaired cortical development leading to neuropsychiatric disorders in adulthood [51]. Furthermore, our SPIED/CMAP analysis predicts that further specificity could be achieved by combinatorial approaches, most notably promoting adult neurogenesis using AR-A014418 combined with adiphenine, a nACh inhibitor that was the most highly ranked pro-rejuvenating and anti-oligodendrogenesis small molecules. Conversely, specific stimulation of oligodendrogenesis could be achieved by LY-294002 in combination with the HDAC inhibitor trichostatin-A, which was the most highly ranked pro-oligodendrogenesis and anti-neurogenic small molecule. Moreover, our analyses highlighted a number of small molecules that are predicted to have an “anti-dorsalizing” or “ventralizing” effect, hence they would be specifically pro-neurogenic. This is of considerable translational interest, because the more ventral SVZ is more developed in humans [52], and adult neurogenesis has recently been described in the striatum [53], which is more extended than in rodents [54]. Moreover, the ventral SVZ gives rise to specific granule cells, such as those forming the Islands of Calleja [55], and our analyses identifies small molecules that may target the different factors that drive the differentiation of such specific neurons. In conclusion, our study establishes unequivocally the effectiveness of employing pharmacogenomic approaches for generating a robust framework that guides mechanistic experiments to manipulate neurogenic niches in the brain, which has considerable potential for identifying therapeutics for neurodegenerative and demyelinating diseases.

## Materials and methods

Unless stated, all materials were purchased from Sigma-Aldrich. All procedures were in accordance and approvals of the United Kingdom Home Office Animals Scientific Procedures Act (1986), Ethics Committee of the Veterinary Department of the Canton of Zurich (Approval ID 182/2011). Experiments in France were performed in accordance with European requirements 2010/63/UE and have been approved by the Animal Care and Use Committee CELYNE (APAFIS#187 & 188). Animal procedures were executed in accordance with UK/Swiss/French law, with strict consideration given to the care and use of animals. All mice were bred over wild-type C57/BL6 background for several generations, and positive animals for Mash1-EGFP were selected at birth under UV light.

### Bioinformatics

Whole genome transcriptome datasets of the isolated SVZ microdomains and region-specific NSCs and TAPs are described in detail in a recent study that described transcriptional regulators acting in SVZ regionalization [1]. Briefly, the rostral periventricular regions of postnatal mice of different ages (P4, P8, and P11) of the Ascl1-EGFP^Bac^ transgenic reporter mouse line were carefully microdissected under a fluorescent binocular microscope in RNAse-free and sterile conditions. Isolated SVZ microdomains were derived from brain coordinates of +1 and 0 relative to Bregma. The Hes5-EGFP reporter mouse line was used in combination with Prominin-1 immunodetection to isolate NSCs from microdissected dorsal and lateral microdomains by fluorescence-activated cell sorting. Similarly, the Ascl1-EGFP^Bac^ transgenic reporter mouse line was used to isolate the 25% brightest cells, i.e., corresponding to TAPs, from either microdomain. Half a litter of animals were used to pool for each replicate throughout. In the present study, these datasets (recently made publicly available from NCBI Gene Expression Omnibus [http://www.ncbi.nlm.nih.gov/geo] GEO Series accession number GSE60905) were analyzed using previously applied bioinformatics methods, with only minor modifications [1]. In brief, data were cured (background subtraction, normalization, and summarization) using robust multi-chip analysis (RMA) using the Partek Genomic Suite software package version 6.6 using stringent false discovery rate (FDR) with *p*-values where necessary in the analysis. All data were normalized collectively with datasets from previous studies of isolated NSC, NPs, and glia (i.e., GSE60905, GSE9566, GSE18765) for optimal parameters. Partek was used to assemble affymetrix data and generate hierarchical clustering and gene lists. GO sets were generated using the latest MGI mouse GO datasets via the Broad Institute (http://www.broadinstitute.org/gsea/index.jsp). The numbers of probes that were differentially expressed across the ten samples analyzed (dNSCs, dTAPs, lNSCs, lTAPs, P4 dSVZ, P4 lateral SVZ, P8 dSVZ, P8 lateral SVZ, P11 dSVZ, and P11 lateral SVZ) represented a total of ~37K probe sets within the 10% FDR range. Genego Metacore (https://portal.genego.com/) and GSEA (http://www.broadinstitute.org/gsea/msigdb/index.jsp) were used to filter and select for probes associated as secreted morphogens (tropic factors, growth factors, extracellular signaling molecules, mitogens, and secreted inhibitors of signaling pathways). The numbers of morphogen from this filtered list that were significantly altered amounted to 530 probes, representing approximately 330 individual genes. Identification of spatially enriched signaling ligands, regardless of sample type (Fig 1A and 1B), was done by comparing all dorsal versus all lateral samples. This gene list was uploaded onto Genego Metacore and Process Network option selected using the default parameters. Determination of the spatial expression profiles of secreted signaling factors in SVZ microdomains (Fig 1C–1H) was performed by comparing datasets using appropriate fold changes and FDR cut-offs (Partek, 1.65-fold change and FDR <5%). For all analyses, raw expression values are provided and Heatmaps are presented in the manuscript.

### SPIED analysis

For SPIED identification of small molecules, the “dorsal NSCs/TAPs,” “lateral NSCs/TAPs,” “postnatal NSCS/TAPs,” “oligodendroglial lineage,” and the “rejuvenating” transcriptional signatures were defined using Partek (1.8-fold change, FDR < 5%), as follows. Probes significant across multiple normalized datasets in the background (representing ~40K probes) were processed. For identification of “dorsalizing” small molecules, dNSCs/dTAPs datasets (positive range) were compared with probes significantly different in the background. This list was then further refined and compared with other postnatal datasets (negative range), using the advanced tab in list manager followed by criteria configuration in generating lists with merged expression profiles. For identification of “ventralizing” small molecules, lNSCs/lTAPs datasets (positive range) were compared as above against with probes significantly different in the background. This list was further refined and compared with other postnatal datasets from the same study (negative range) [1]. For identification of “pro-oligodendrogenic” small molecules, publicly available datasets of forebrain-derived OL lineage cells (positive range; GSE9566) were compared to dNSCs and dTAPs (negative range) from which they emerge. Finally, for identification of “rejuvenating” small molecules, publicly available datasets of adult NSCs [29] (positive range) were compared with postnatal dNSCs/lNSCs (negative range) [1]. These expression profiles, consisting of gene symbols of “enriched” genes, were next uploaded onto SPIED (http://www.spied.org.uk/cgi-bin/HGNC-SPIED3.1.cgi) to interrogate the CMAP initiative in an unbiased way and identify small molecules predicted to promote the positive ranges of gene signatures using default parameters. The Broad CMAP 2.0 (CMAP2.0) database consists of the transcriptional profiles corresponding to the effects of small molecules at various concentrations and treatment times using panels of human cell lines. The data are available for download in the form of ranked probe sets for each microarray sample on the [HG-U133A] Affymetrix Human Genome U133A Array platform.

Identified small molecules cellular targets were exhaustively characterized using publicly available drug repositories (www.DrugBank.ca/; www.genome.jp/kegg/drug/; http://insilico.charite.de/supertarget/; www.pharmgkb.org; http://stitch.embl.de/). Small molecules protein targets identified were cross-checked in http://www.genecards.org/ for classifying them under general GO terms. All analyses presented in Fig 2 are shown as a percentage.

### Small molecule target gene analysis

“Target genes” are defined as the genes from the queried expression profiles that are also induced by a given small molecule. Target genes for each analysis were generated as follows. To generate lists of genes perturbed by the small molecules, gene replicates were pooled and the relative expression levels calculated. Changes passing the Student’s *t* test *p*-value of ≤0.05 were processed, and when there were multiple probes for a given gene, the probe with the biggest fold change was assigned to the gene. These were aligned for matching signatures with the transcriptional profiles corresponding to the small molecules repurposed in the CMAP using pattern-matching algorithms that enable identification of functional connections between drugs, genes, and diseases through the transitory feature of common gene-expression changes [4]. The entire database is available for download (http://www.broadinstitute.org/gsea/msigdb/index.jsp) in the form of ranked probe sets for each microarray sample on the [HG-U133A] Affymetrix Human Genome U133A Array platform.

For analysis of “target genes” of select small molecules, we first performed hierarchical clustering of their expression profile in the various cell types and lineage that compose the SVZ using the following datasets: purified postnatal NSCs and TAPs (GSE60905 [1]), for purified glial cells (GSE9566 [25]), and adult NSCs/NPs/ependymal cells (GSE18765 [29]). Target genes were then classified by “Process network and pathway maps” GO categories. Briefly, target gene lists containing the contrasts and fold changes were analyzed via the web platform http://www.broadinstitute.org/cmap/index.jsp and functionally classified using Genego Metacore (https://portal.genego.com/) for Process Networks and Pathway Maps.

Last, target genes were studied further for obtaining the shortest path between genes associated with highly ranked process networks and pathway maps using standard Dijkstra’s shortest paths algorithm and applying default parameters [7,56]. Background RMA normalized data for all probe sets relevant for the postnatal and adult SVZ derived from postnatal NSCs and TAPs ((GSE60905) [1]), purified glial cells ((GSE9566 [25]), and adult NSCs/NPs/ependymal cells ((GSE18765 [29]) were uploaded onto Genego Metacore (raw data are provided with the manuscript). This allows obtaining, visualizing, aligning, and clustering the most relevant target genes based on small molecule target genes with reference to basally expressed genes in the SVZ. Objects within this network were restricted to 70–80 in accordance with those highly ranked and most significant within the earlier GO analysis, and signaling-to-transcriptional options were selected. Internal clusters (2–4) within the network module were arranged according to the highest ranked GO pathways within the analysis in the pathway selection menu. A full description of the definition of objects and nodes can be found here: https://portal.genego.com/legends/MetaCoreQuickReferenceGuide.pdf.

### SVZ microdissection, qPCR, and western blot

The SVZ microdomains were microdissected using previously published protocols [1]. In brief, mouse pups were killed humanely by cervical dislocation. In sterile and RNAse-free conditions, brains were rapidly dissected free and placed in ice-cold postnatal-specific coronal brain matrix (Zivic Instruments, US) to obtain tissue segments of 500-μm thickness containing the rostral periventricular tissue as above for whole genome transcriptome analysis. For examination of LY-294002-induced genes by qPCR, pups were treated by intraventricular infusion (see below) at P9 and P10, and 180 min following final infusion, tissue was microdissected. Five pups were used to pool for individual “n” numbers, and RNA was systematically amplified for all “n” numbers as previous [10]. For western blot (S1A Fig), pups aged at P10 were treated with LY-294002 (see below), and 45 min following injection, pups were systematically killed by cervical dislocation and tissue microdissected and flash frozen in lysis buffer in liquid nitrogen for storage at −80°C [10]. One litter of pups was pooled to yield 1 “n” number. For qPCR experiments, relative gene expression was determined using the 2 ^ΔΔ-^CT method versus the housekeeping gene GAPDH (Glyceraldehyde-3-phosphate dehydrogenase). See S7 Table for a list of primers used in the study. Primers were designed by Primer Express 1.5 software and synthesized by Eurofins (Ebersberg, Germany). Unstated primers in main text were custom designed and obtained from (Qiagen).

Protein was extracted with lysis buffer and standard procedures as previous [57]. For SDS-PAGE gels, 15 μg was loaded and transferred to a PVDF membrane (GE Healthcare, Amersham). Blots were preincubated in a blocking solution of 5% BSA in 0.2% TBST (0.1 M Tris base, 0.1% Tween 20, pH 7.4) for 1 h at RT and incubated with primary antibodies overnight at 4°C and after washing, with a horseradish peroxidase-conjugated anti-rabbit antibody (1:10,000–1:25,000; Pierce Biotechnology). Primary antibodies were all obtained from Cell Signaling and used in concentrations of 1:500 for phosphor-forms and 1:2000 for total forms of protein. Protein bands were detected by adding SuperSignal West Pico Chemiluminescent Substrate (Pierce) by exposing the blot in a Stella detector (Raytest). Densitometry analysis was performed with NIH software and by normalizing the band intensities to total Akt or total Erk1/2 values. Intensity values for pAkt were combined and pAkt-473 shown only.

Gene expression and western blot data are presented as mean + standard deviation of the mean (SD) or standard error of the mean (SEM), respectively, and samples compared for significance using unpaired *t* test (*t* test) or (Prism v3.02 software; GraphPad).

### In vivo procedures

Animals were killed humanely by cervical dislocation and brains removed rapidly to ice-cold fixative. Mouse pups of similar size were used throughout. Mice aged P8 were treated by intraventricular infusion into the LV daily for 3 d, and brains sampled at P11, overnight following the final injection. Mice were deeply anesthetized under isofluorane and differing concentrations of LY-294002 (Sigma-Aldrich), dissolved in sterile DMSO, sterile filtered, and co-administered with sterile saline delivered into the CSF of the LV using a Hamilton syringe at point 2 mm from the midline along the Bregma and to a depth of 2 mm. Sterile saline/DMSO vehicle were used as controls throughout this study. Concentrations of small molecules infused into the CSF of the lateral ventricle were based on the known CSF availability of agents, as performed previously by the authors [57] and elsewhere based on CSF volume and turnover in the adult mouse [58]. Methods applied for studying postnatal oligodendrogenesis are based on previous studies using C57/BL6 mice and transgenic mouse line in which fluorescent reporters DsRed are under control of the PLP promoter [57,59].

Methods applied for studying rejuvenation of the adult SVZ were perform by infusing GSK3β inhibitors into the ventricular system of adult P90 mice. Animals were anesthetized with a subcutaneous injection of Ketamin (60 mg/kg body weight), Xylazine (13 mg/kg body weight), and Acepromazine (1.5 mg/kg body weight) before being fixed in a stereotaxic apparatus. After exposure of the skull surface, a canula (Alzet, Brain infusion kit 3) was stable implanted at the following coordinates (Bregma −0.5 mm; lateral 1 mm, depth: 2.5 mm) for intraventricular infusion of the GSK3β inhibitors CHIR99021 and AR-A014418. Delivering the small molecules was achieved over a period of 3 d using an osmotic miniupump (1 μl/h, model 1003D; Alzet Osmotic Pumps) into the CSF. Sham animals received all surgical steps, catheter implantation, and pump insertion.

For studying recruitment of SVZ NSCs following hypoxia and small molecule administration, dSVZ NSCs were permanently labeled by dorsal electroporation [60] of a Cre plasmid (Cambridge, MA, www.addgene.org, plasmids 13775) in Cre-reporter mice (ROSA26-Flox-Stop-Flox YFP, Jackson Laboratories). A pCAGs-Cre plasmid under a chicken β-actin promoter was obtained from Addgene (Cambridge, MA, www.addgene.org, plasmids 13775). Mice aged P1 were dorsally electroporated, then placed in a hypoxic rearing chamber maintained at 9.5%–10.5% O_2_ concentration by displacement with N_2_ as described previously [43]. Hypoxia began at P3 for 8 d until P11. A separate group was maintained in a normal atmosphere (normoxic group). CHIR99021 was administered by intranasal administration as previously described [45]. Mucus was first permeabilized by the use of type IV hyaluronidase, then, 10 μl of CHIR99021 (Sigma) was administrated 4 times (starting at the end of the hypoxic period, then every 12 h), at a concentration of 1.5 mM in sterile PBS (Vehicle was used as a control). Mice were killed 8 d after cessation of hypoxia at P19.

### Immunohistochemistry

Standard immunofluorescence protocols were applied as previously described [15]. Mice were killed by injection with an intraperitoneal overdose of pentobarbital (Eutha77 in Ringer’s solution) followed by transcardial perfusion with 4% paraformaldehyde (PFA) dissolved in 0.1 M phosphate buffered saline (PBS; pH 7.4). Following removal, brains were post-fixed in 4% PFA overnight at 4°C and cut in coronal sections at 30–50 μm thickness for obtaining serial sections. Primary antibodies used were goat anti-Dcx (1:400 Santa Cruz); anti-Mcm2 (1:400 Santa Cruz); chicken anti-β-galactosidase (1:500, Abcam); mouse anti-Ki67 (1:500, BD Pharmingen); (1:300, Millipore); rabbit anti-GFAP (1:300, DAKO); rabbit anti-Olig2 (1:400, Millipore); chicken anti-GFP (1:1000; Abcam, AB13970); mouse anti-NeuN (1:500, Millipore, MAB377); rat anti-MBP (1:300, Millipore, AB40390); rabbit anti-Tbr2 (1:500, Abcam). Appropriate secondary antibodies conjugated with Alexafluor 488, 568, or 405 (1:400, Molecular Probes) were applied. Control experiments were performed using appropriate blocking peptides where available or otherwise by omission of the primary antibody. Fluorescent labeling of cells in S-phase by EdU (5-ethynyl-2′-deoxyuridine) detection was performed following manufacturers guidelines using Click-it EdU Alexa Fluor 555 imaging kit (Invitrogen). Tissues were mounted on poly-lysine coated glass slides with Vectashield mounting media (Vector Laboratories) and sealed with coverslips.

### Imaging and quantification procedures

Imaging and analysis methods are described in detail in our previous methodological study [61]. All quantifications were performed using a homogenous sampling approach that has been optimized for three-dimensional analysis of microdomains in the mouse SVZ [10] and provides an accurate quantification equivalent to exhaustive stereological methods from which it is adapted [61]. In brief, serial coronal sections were processed throughout the entire rostro-caudal extent of the SVZ (series of six sections for adult tissues and at least three for the postnatal SVZ). Quantification was performed on equivalent areas in each experimental group [10]. Images were captured using a Zeiss LSM Meta 5.1, Zeiss LSM Meta 7.1, or Leica SPEII confocal microscope and processed with Zeiss LSM Image Examiner (V. 5.2.0.121) or LAS-AF software (V. 2.7), maintaining the acquisition parameters constant to allow comparison between samples. The number of cells expressing the markers Ki67, EdU, Olig2, Tbr2, Mcm2, Dcx, Ascl1, and GFAP were quantified with at least three fields of view per section on series of equally spaced sections of 40-μm thickness encompassing the entire rostral lateral ventricle as previously described [10,59]. The dSVZ in both ages was defined based on DAPI counterstaining (Invitrogen). Quantifications were performed on confocal *z*-stacks of 230 μm^2^ × 230 μm^2^ in the *x*-*y*-plane and 30 μm in the *z*-plane, with a volume of 1.6 × 10^6^ μm^3^. For the case of GFAP+ cells, i.e., NSCs, only cells directly adjacent to the ependymal layer were analyzed as previous for similar postnatal ages [10]. The myelin index (MI), a means to measure postnatal myelination in the corpus callosum, was done in serial sections from PLP-DsRed mice [59]. The number of myelin sheaths crossing a diagonal transect was counted in each confocal *z*-section at 1, 5, 10, 15, 20, 25, and 30 μm (captured using a 40× objective) so that the MI represents the density of DsRed+ myelin sheaths within a volume of 1.6 × 10^6^ μm^3^. Statistical significance was tested using GraphPad Prism v302 for multiple variables, using or one-way analysis of variance (ANOVA) followed by Bonferroni’s post hoc test, and for two variables, using unpaired *t* tests (referred to as *t* test), where appropriate.

## Supporting information

S1 Fig LY-294004 inhibits PI3K/Akt signaling, modulates expression of transcripts, promotes specifically Olig2+ progenitors and augments myelination. . **A)** P9 mice were treated with 0.06 mM LY-294002 and saline/DMSO as controls and SVZ microdomains were analyzed by western blot 45 mins after infusion. Representative immunoblots and mean densitometric values for protein levels (±SEM, n = 3 for control and LY-294002), for total-Akt and pAkt and total Erk1/2 and pErk1/2. Significance was tested by t test. **B)** qPCR was performed on microdissected dorsal SVZ 180 min following final infusion to detect cell specific transcripts in earlier NSC/NP lineages, glial lineages, secreted trophic factors, and signaling pathway components or target genes. Data’s are expressed as the mean (± SD; n = 3 for control and LY-294002) % change of relative expression values, GAPDH normalized. *p<0.05; **p<0.01, ***p<0.001; *t* tests. White bars indicate controls and black bars for LY-294002. **C)** P8 PLP-DsRed transgenic mice (for identifying OLs and myelin sheaths) were treated daily with 0.06 mM LY-294002 and saline/DMSO as controls for 3 days and sacrificed at P11 for immunolabelling with MBP for changes in myelination. Histogram of the myelin index in the corpus callosum; data are mean number of myelin sheaths ± SEM (n = 4 for control and LY-294002) in a constant volume and were tested for significance using unpaired t test (** p<0.01). Confocal micrographs show enhanced MBP immunolabelling following LY-294002 and insets show LY-294002 induced PLP-DsRed+ OLs support more normal appearing myelin sheaths. Right panels show enhanced DsRed expression and greater densities in PLP-DsRed+ OLs. Images are flattened confocal z-stacks of thickness 15 μm (left panels; captions are single z-sections), or 10 μm thickness in right panels. Scale bar in right panels = 25 μm; in left panels (5 μm in captions) and 15 μm in left single channel panels.
(TIF)

S2 Fig Confirmation in the decline in SVZ activity, Wnt-signaling and dorsal-derived lineages. The spatiotemporal gene expression changes in microdissected SVZ microdomains were examined by qPCR of selected genes and processed in Partek Genomics Suit 6.6 and presented as an intensity heatmap. Only transcripts that passed the criteria of p<0.05 ANOVA versus its adjacent (dorsal versus lateral) or temporal (2.5 months (n = 4), 6 months (n = 3) and 1 year (n = 3)) are presented.
(TIF)

S3 Fig GSK3β inhibitors AR-A014418 and CHIR99021 successfully activate Wnt/β-catenin signaling *in vitro* and *in vivo*. **A)** GSK3β inhibitors (AR-A014418 and CHIR99021) activate the Wnt canonical pathway *in vitro*, as indicated by increased immunodetection of Ser9-GSK3β phosphorylation. Graph shows the quantification of optical density and n ≥ 75 cells analyzed per group. Images illustrate the experimental conditions. Scale Bar = 40 μm. **B)** qPCR analysis of Wnt signaling target genes expression *Axin2*, *Lef1* and *Tcf* and the Shh signaling target gene *Gli1* in the dorsal SVZ following subcutaneous injections of CHIR99021 (500μM), as previously observed for AR-A014418 [10]. Error bars represent standard error mean (SEM). **, p<.01; *, p<.05; t test.
(TIF)

S4 Fig Sub-cutaneous injections of AR-A014418 and CHIR99021 increase glutamatergic neuron and OL progenitor numbers in the early postnatal dorsal SVZ. AR-A014418, CHIR99021 or a vehicle (CTR) was injected subcutaneously during 2 days before isolation of the brain. **A)** qPCR analysis of the dorsal SVZ markers *Tbr2* and *Pax6* in the dorsal SVZ following subcutaneous injections of CHIR99021 (500μM). **B)** Representative picture of EDU and Tbr2 stainings in the dorsal SVZ. Scale Bar = 100 μm (overview) and 40 μm (right panels). **C)** Percentage increase of Tbr2+, Olig2+ and Ki67+ cells in the dorsal SVZ after AR-A014418 and CHIR99021 administration at different concentration. Values are normalized compared to the controls. Error bars represent standard error mean (SEM) and n = 7 for control and 3 n numbers for each GSK3β inhibitor group. **, p<0.01; *, p<0.05; t test.
(TIF)

S5 Fig Procedure for intraventricular infusion of GSK3β inhibitors in P90 mice. **A**) Infusion site (red arrow), on caudal coronal section from Mouse Paxinos Atlas. Here DAPI (in red) is infused to visualize the pattern of diffusion from the cerebrospinal fluid, on a coronal section stained with Nissl. **B**) Note that the rostral regions of the lateral ventricles, where quantifications were performed remained intact.
(TIF)

S1 Table List of dorsalizing small molecules (Associated with Table 1A). Gene lists (dNSCs/dTAPs were compared with their ventral counterparts and the P4 SVZ (see Materials and Methods) were uploaded onto www.spied.org.uk to perform the CMAP small molecule analysis. Agents with a positive correlation have the ability to promote genes associated postnatal dorsal lineages, whereas those in the negative range have an anti-correlation (i.e. repressive effect) on dNSCs/dTAPs. Small molecules are ranked according to the largest numbers of "target or perturbed genes".
(XLSX)

S2 Table Table of ventralizing small molecules (Associated with Table 1B) . Gene lists (vNSCs/vTAPs were compared with their dorsal counterparts and the P4 adjacent SVZ tissue (see Materials and Methods) were uploaded onto www.spied.org.uk to perform the CMAP small molecule analysis. Agents with a positive correlation have the ability to promote genes associated postnatal ventral lineages, whereas those in the negative range have an anti-correlation (i.e. repressive effect) on vNSCs/vTAPs. Small molecules are ranked according to the largest numbers of "target or perturbed genes".
(XLSX)

S3 Table Table of small molecules to promote oligodendrogenesis (Associated with Table 1C). Gene lists (isolated OL lineage cells at different stages of differentiation were compared against those of dNSCs+dTAPs (see Materials and Methods) were uploaded onto www.spied.org.uk to perform the CMAP small molecule analysis. Agents with a positive correlation have the ability to promote genes associated with the OL lineage, whereas those in the negative range have an anti-correlation (i.e. repressive effect) with the OL lineage. Small molecules are ranked according to the largest numbers of "target or perturbed genes".
(XLSX)

S4 Table List of small molecules to rejuvinate adult NSCs (Associated with Table 1D). Gene lists (dNSCs+vNSCs versus adult NSCs (see Materials and Methods) were uploaded onto www.spied.org.uk to perform the CMAP small molecule analysis. Agents with a positive correlation have the ability to promote genes associated with earlier NSCs, whereas those in the negative range have an anti-correlation (i.e. repressive effect) on earlier NSCs phenotypes. Small molecules are ranked according to the largest numbers of "target or perturbed genes".
(XLSX)

S5 Table CMAP of “LY-294002” induced genes (Associated with Fig 3). Uploaded gene list for generating list of drugs to perturb OLs from postnatal dNSCs/dTAPs were further analysed on the to provide a list of gene targets that are likely to be differentially affected. Also given are relative fold change intensity and p-values.
(XLSX)

S6 Table CMAP of “AR-A014418” induced genes (Associated with Fig 5). Uploaded gene list for generating list of drugs to perturb adult NSC signatures into postnatal NSC signatures were further analysed on the CMAP for AR-A014418 to provide a list of gene targets that are likely to be differentially affected. Also given are relative fold change intensity and p-values.
(XLSX)

S7 Table List of oligonucleotide Primers used. (XLSX)

S1 Data Accompanying raw data used in the manuscript. (XLSX)

## Abbreviations

CMAP: connectivity map
CSF: cerebrospinal fluid
dSVZ: dorsal SVZ
EdU: 5-ethynyl-2 deoxyuridine
FDR: false discovery rate
GFAP: glial fibrillary acidic protein
GO: gene ontology
HDAC: histone deacetylase
LV: lateral ventricle
mACH: muscarinic acetylcholine
NP: neuronal precursor
NSC: neural stem cell
OL: oligodendrocyte
OP: oligodendrocyte precursor
P: postnatal day
PLP: proteolipid protein
qPCR: quantitative PCR
RMS: rostral migratory stream
SPIED: searchable platform-independent expression database
SVZ: subventricular zone
TAP: transient amplifying progenitor
TF: transcription factor
