# Supplementary figures and images for: Pharmacogenomic identification of small molecules for lineage specific manipulation of subventricular zone germinal activity
Source: PLoS Biol. 2017 Mar 28;15(3):e2000698. doi: 10.1371/journal.pbio.2000698 (PMC5370089; doi:10.1371/journal.pbio.2000698)

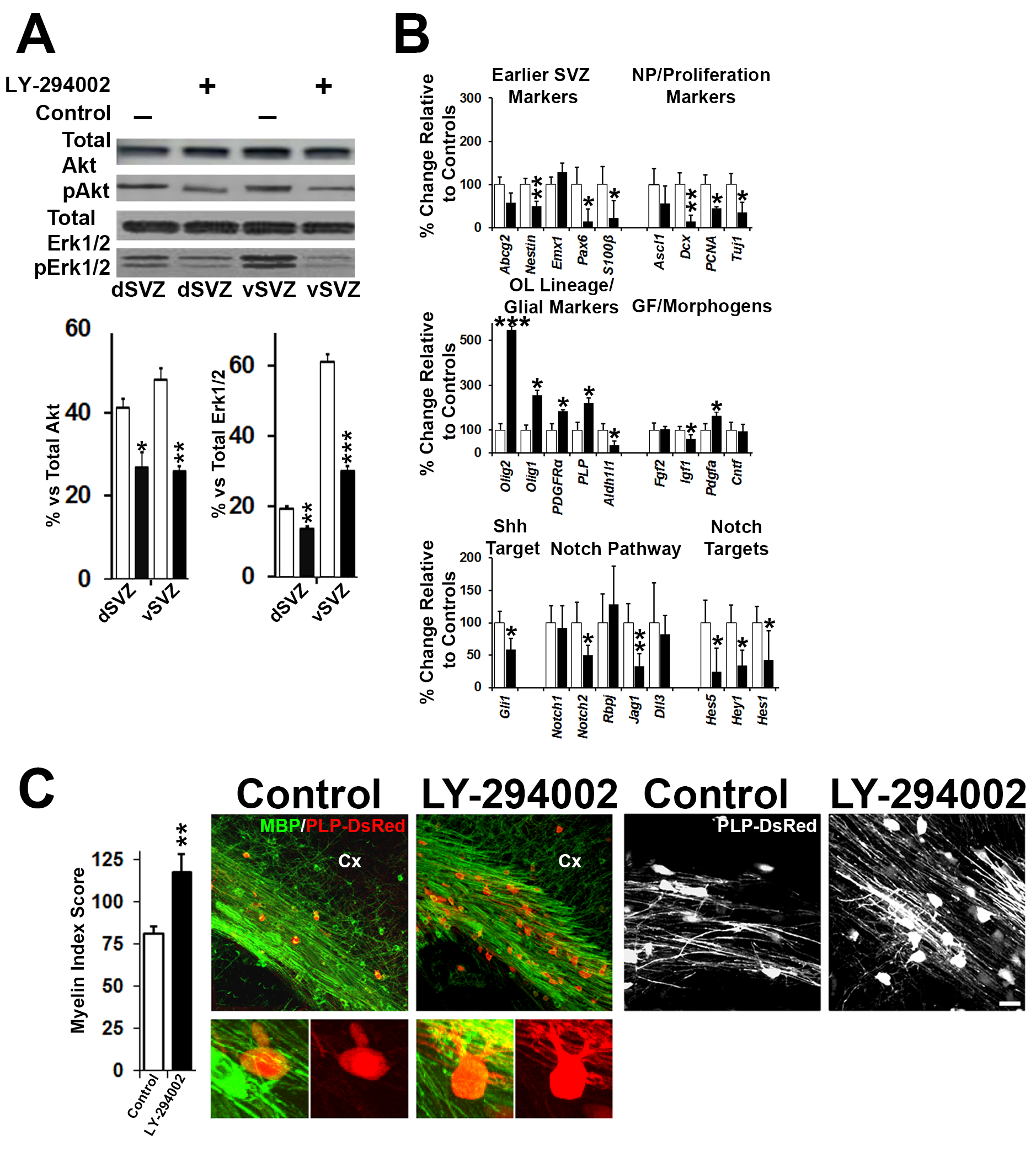

Supplement: S1 Fig — . A) P9 mice were treated with 0.06 mM LY-294002 and saline/DMSO as controls and SVZ microdomains were analyzed by western blot 45 mins after infusion. Representative immunoblots and mean densitometric values for protein levels (±SEM, n = 3 for control and LY-294002), for total-Akt and pAkt and total Erk1/2 and pErk1/2. Significance was tested by t test. B) qPCR was performed on microdissected dorsal SVZ 180 min following final infusion to detect cell specific transcripts in earlier NSC/NP lineages, glial lineages, secreted trophic factors, and signaling pathway components or target genes. Data’s are expressed as the mean (± SD; n = 3 for control and LY-294002) % change of relative expression values, GAPDH normalized. *p<0.05; **p<0.01, ***p<0.001; t tests. White bars indicate controls and black bars for LY-294002. C) P8 PLP-DsRed transgenic mice (for identifying OLs and myelin sheaths) were treated daily with 0.06 mM LY-294002 and saline/DMSO as controls for 3 days and sacrificed at P11 for immunolabelling with MBP for changes in myelination. Histogram of the myelin index in the corpus callosum; data are mean number of myelin sheaths ± SEM (n = 4 for control and LY-294002) in a constant volume and were tested for significance using unpaired t test (** p<0.01). Confocal micrographs show enhanced MBP immunolabelling following LY-294002 and insets show LY-294002 induced PLP-DsRed+ OLs support more normal appearing myelin sheaths. Right panels show enhanced DsRed expression and greater densities in PLP-DsRed+ OLs. Images are flattened confocal z-stacks of thickness 15 μm (left panels; captions are single z-sections), or 10 μm thickness in right panels. Scale bar in right panels = 25 μm; in left panels (5 μm in captions) and 15 μm in left single channel panels. (TIF) [file pbio.2000698.s001.tif]

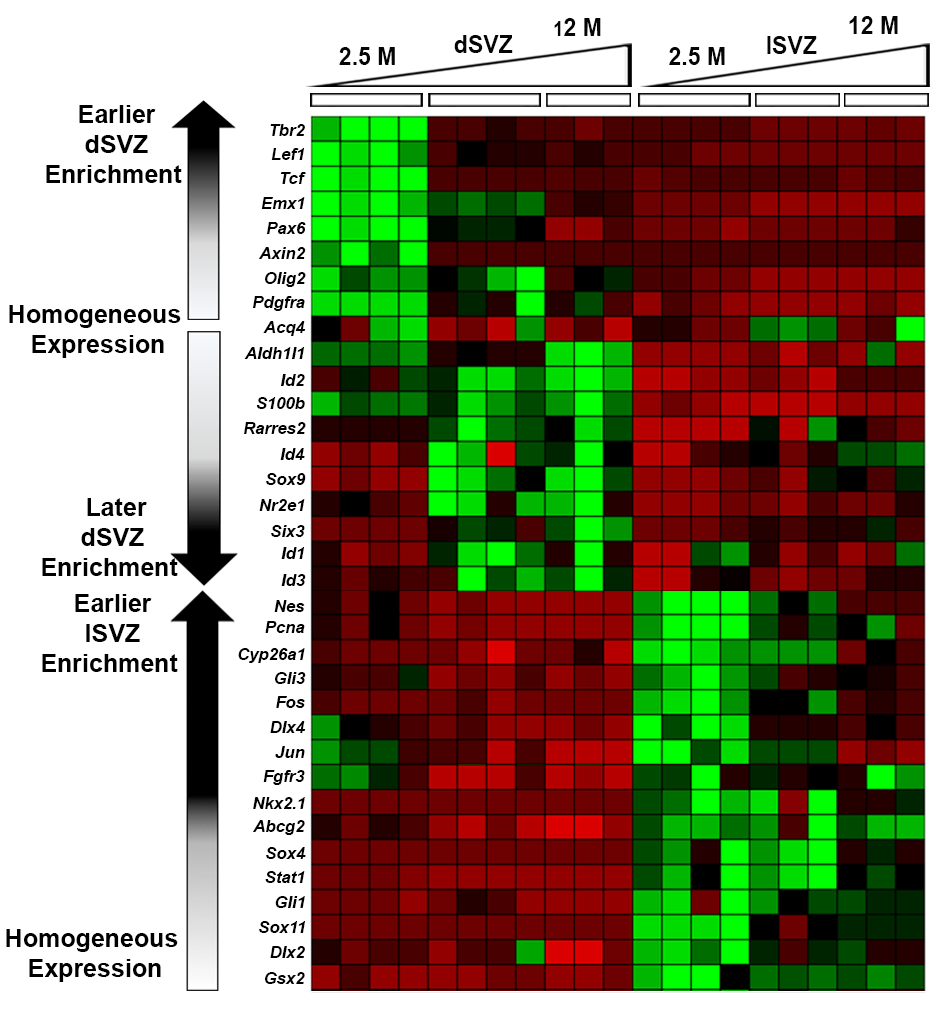

Supplement: S2 Fig — The spatiotemporal gene expression changes in microdissected SVZ microdomains were examined by qPCR of selected genes and processed in Partek Genomics Suit 6.6 and presented as an intensity heatmap. Only transcripts that passed the criteria of p<0.05 ANOVA versus its adjacent (dorsal versus lateral) or temporal (2.5 months (n = 4), 6 months (n = 3) and 1 year (n = 3)) are presented. (TIF) [file pbio.2000698.s002.tif]

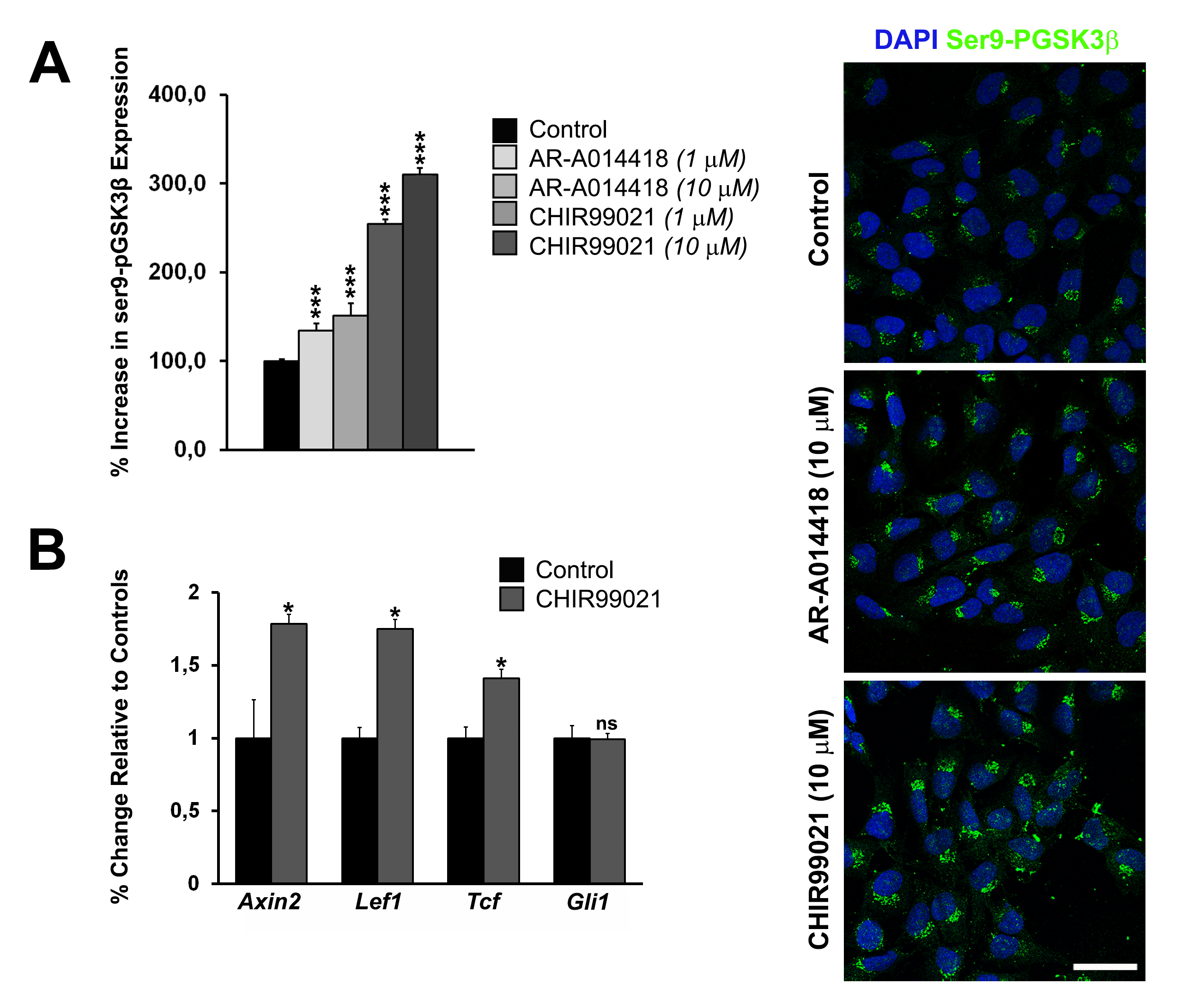

Supplement: S3 Fig — A) GSK3β inhibitors (AR-A014418 and CHIR99021) activate the Wnt canonical pathway in vitro, as indicated by increased immunodetection of Ser9-GSK3β phosphorylation. Graph shows the quantification of optical density and n ≥ 75 cells analyzed per group. Images illustrate the experimental conditions. Scale Bar = 40 μm. B) qPCR analysis of Wnt signaling target genes expression Axin2, Lef1 and Tcf and the Shh signaling target gene Gli1 in the dorsal SVZ following subcutaneous injections of CHIR99021 (500μM), as previously observed for AR-A014418 [10]. Error bars represent standard error mean (SEM). **, p<.01; *, p<.05; t test. (TIF) [file pbio.2000698.s003.tif]

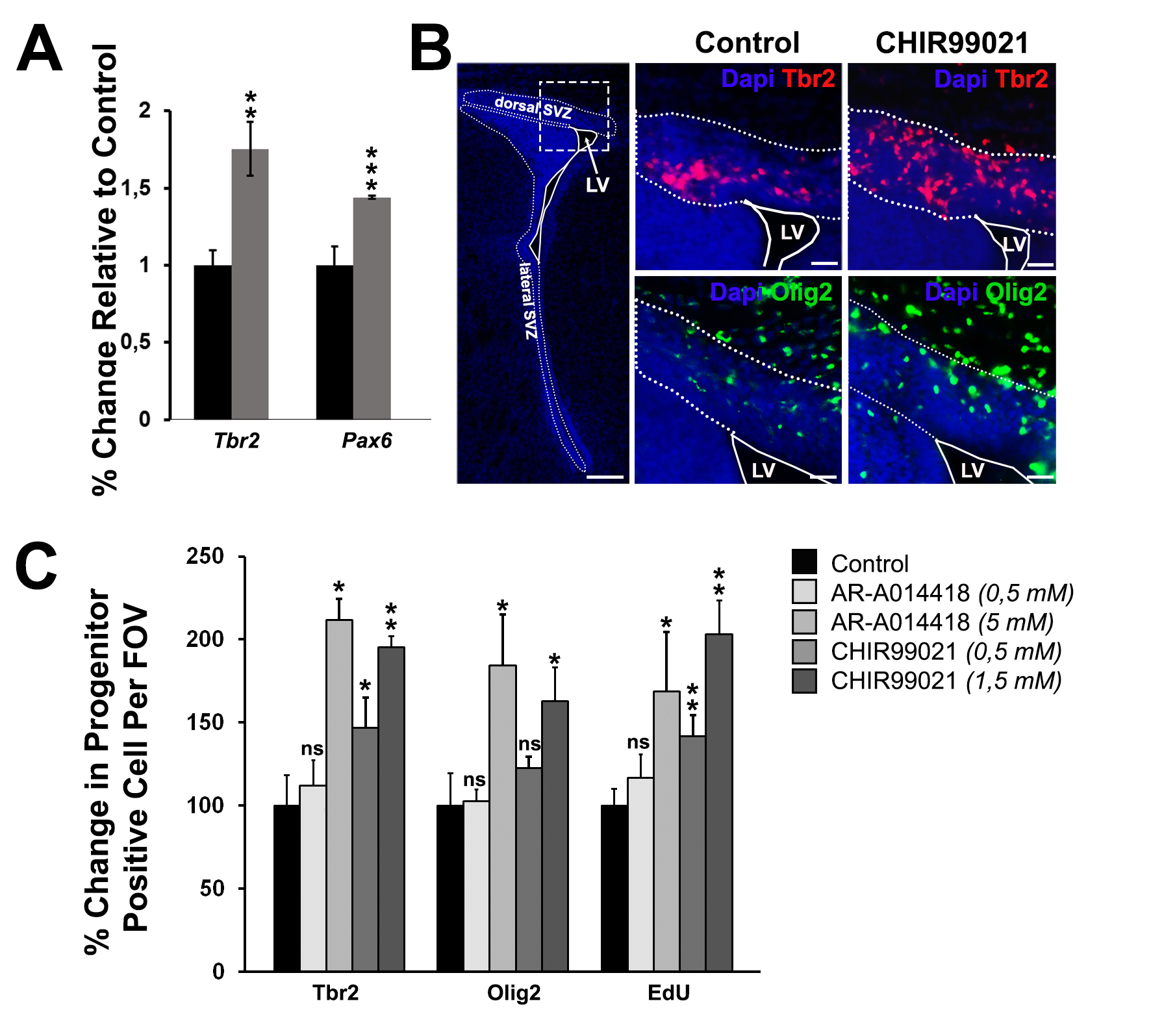

Supplement: S4 Fig — AR-A014418, CHIR99021 or a vehicle (CTR) was injected subcutaneously during 2 days before isolation of the brain. A) qPCR analysis of the dorsal SVZ markers Tbr2 and Pax6 in the dorsal SVZ following subcutaneous injections of CHIR99021 (500μM). B) Representative picture of EDU and Tbr2 stainings in the dorsal SVZ. Scale Bar = 100 μm (overview) and 40 μm (right panels). C) Percentage increase of Tbr2+, Olig2+ and Ki67+ cells in the dorsal SVZ after AR-A014418 and CHIR99021 administration at different concentration. Values are normalized compared to the controls. Error bars represent standard error mean (SEM) and n = 7 for control and 3 n numbers for each GSK3β inhibitor group. **, p<0.01; *, p<0.05; t test. (TIF) [file pbio.2000698.s004.tif]

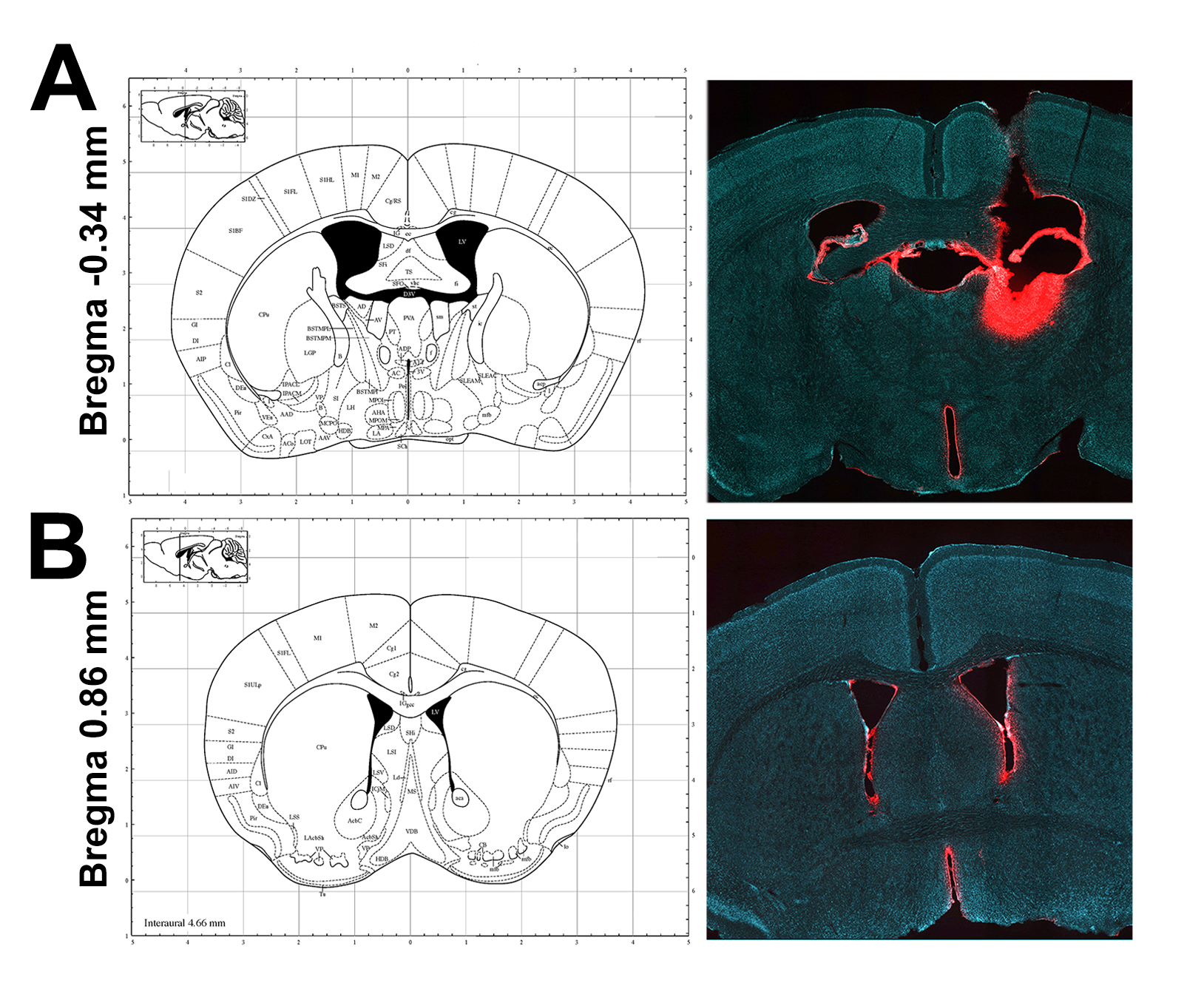

Supplement: S5 Fig — A) Infusion site (red arrow), on caudal coronal section from Mouse Paxinos Atlas. Here DAPI (in red) is infused to visualize the pattern of diffusion from the cerebrospinal fluid, on a coronal section stained with Nissl. B) Note that the rostral regions of the lateral ventricles, where quantifications were performed remained intact. (TIF) [file pbio.2000698.s005.tif]
